# Otology

**Published:** 1896-12

**Authors:** W. H. Harsant


### OTOLOGY.

**Cerebral abscess following middle-ear disease** is almost always the result of a *chronic* suppuration; so that we are generally able to assure a patient with recent acute suppuration, no matter how severe, that brain abscess is not likely to occur. Politzer says: "The otitic cerebral abscess usually develops as the result of chronic suppuration of the middle ear, very seldom in the course of acute purulent inflammation of the middle ear."[1] Gowers also remarks that, "The ear disease [which causes brain abscess] is usually chronic, and has existed for several years—five, ten, fifteen, and even twenty or twenty-five years—before it caused the abscess; very rarely the mischief has existed only for a few weeks or months."[2]

This being the case, it is interesting to notice that two cases of brain abscess following *acute* suppuration of the middle ear have been published during the last half-year.

The first, reported by Milligan[3], is a case of temporo-sphenoidal abscess secondary to acute left-sided suppurative middle-ear disease, treated by operation, but followed by acute hernia cerebri and death. The patient was a man aged forty-eight, who had enjoyed good health up to March, 1895, when he contracted a severe attack of influenza. He was laid up for a few weeks, after which he returned to his work—that of foreign

[1] *Text-book of Diseases of the Ear*, 1894.
[2] *Manual of Diseases of the Nervous System*, 2nd Edition, vol. ii. 1893, p. 474.
[3] *Arch. Otol.*, 1896, xxv. 265.

correspondent to a large commercial firm—and remained on duty until September of the same year. About this time his left ear became suddenly painful, and a profuse purulent discharge made its appearance. He was troubled with constant pulsating tinnitus, and with a marked degree of deafness. For three months he remained under the care of his ordinary medical attendant, during which time various forms of local treatment were tried. Pain in and around the ear and over the corresponding side of the head was so continuous and severe as to practically incapacitate him. When first seen by Dr. Milligan upon December 6, 1895, the following notes were made: "The patient complains of intense pain in the head, especially over the frontal region. This pain is aggravated by any movement of the head and especially by percussion. His mental condition is distinctly apathetic and his cerebration is slow and sluggish. He exhibits in a marked degree the condition of sensory aphasia or word-deafness, while, in addition, motor aphasia is present, although to a less marked degree. There is extreme ptosis, and the left pupil is dilated. The left optic disc is swollen and its edges are fluffy. There is left facial paralysis, but no apparent paresis of arm or leg . . . The temperature is 98.8° F., the pulse 66. During the preceding few days his wife says that there has been incontinence of urine and fæces. She also states that for a week he has had hardly any sleep on account of the severe pain in his head, which he constantly clutches between his hands and complains of." The skull was trephined over the temporo-sphenoidal lobe, and an abscess containing four drachms of perfectly odourless pus was evacuted. The abscess was drained, and all went well until the end of January, when a hernia cerebri formed, and the patient died of advancing basal meningitis.

The other was a case of acute otitis media, followed by an abscess in the temporo-sphenoidal lobe. Operation ; death from shock. It is reported, with two photographs, by Dr. Gorham Bacon.[1] The patient was a young man of twenty-five years. Eight weeks before being seen he had a slight discharge from the left ear, but before this he never had any ear disease. One month ago he had been seen by a surgeon, who found on examination that the seat of the trouble was in the attic, which was congested and bulging. This was incised, and a few drops of pus were evacuated. Severe headache followed, with tenderness to pressure over the mastoid. For three weeks he has had loss of memory for objects and names of friends. Memory for events good. He has been extremely irritable when suffering from headache, and his friends have noticed that he has acted and talked "queerly." On examination there was very slight discharge in the left canal, but there is bulging of Shrapnell's membrane, with a small perforation. He is unable to name certain objects held before him, such as "scarf-pin," "cuff button," &c. No motor aphasia. Temperature 98.8°, pulse full

[1] *Arch. Otol.*, 1896, xxv. 249.

and slow 56, respirations 16. Heart shows long systolic murmur, A brain abscess was suspected, but the antrum was first opened and washed out. This seemed to give relief for the first week, but then pain in the head reappeared, and he had spells of hilarity, *i.e.* laughing and singing. Vomiting came on, and then delirium, followed by somnolence. The skull was then trephined over the temporo-sphenoidal lobe, and an abscess cavity found containing half an ounce of pus. This was evacuated, and the cavity packed with iodoform gauze. The patient died from shock two hours after operation.

W. H. HARSANT.

